# Vitamin D receptor Bsm I polymorphism and osteoporosis risk in postmenopausal women: a meta-analysis from 42 studies

**DOI:** 10.1186/s12263-020-00679-9

**Published:** 2020-11-25

**Authors:** Jun Long Liao, Qiang Qin, Yong Sheng Zhou, Ru Ping Ma, He Chao Zhou, Mao Rong Gu, Yun Ping Feng, Bo Yuan Wang, Ling Yang

**Affiliations:** 1 The People’s Hospital of Yuxi City, The 6th Affiliated Hospital of Kunming Medical University, Yuxi, 653100 China; 2 The Sports Department, Zhejiang University of Science & Technology, Hangzhou, 310023 China; 3 The Key Lab of Sports and Rehabilitation, Faculty of Physical Education, Yuxi Normal University, Yuxi, 653100 China

**Keywords:** Vitamin D receptor, BsmI polymorphism, Osteoporosis, Postmenopausal, Meta-analysis

## Abstract

**Objective:**

This study aimed to quantitatively summarize the evidence for VDR BsmI gene polymorphism and osteoporosis risk in postmenopausal women.

**Materials and methods:**

The PubMed, EMBASE, Weipu, CNKI, and Wanfang databases were searched for eligible studies. Case-control studies containing available genotype frequencies of B/b were chosen, and odds ratio (OR) with 95% confidence interval (CI) was used to assess the strength of this association.

**Results:**

4485 osteoporosis and 5490 controls were identified in our meta-analysis. In the stratified analysis, a significant association was observed between VDR BsmI gene polymorphism and osteoporosis susceptibility in Caucasians (additive model: OR = 0.809, 95% CI 0.678~0.965, *p* = 0.019; recessive model: OR = 0.736, 95% CI 0.568~0.955, *p* = 0.021; and co-dominant model: bb vs. BB OR = 0.701, 95% CI 0.511~0.962 *p* = 0.028), and we failed to find any significant relationship in Asians.

**Conclusion:**

The present meta-analysis suggests that VDR BsmI genotype is associated with increased risk of postmenopausal osteoporosis in Caucasians but not in Asians. To draw comprehensive and true conclusions, further prospective studies with larger numbers of participants worldwide are needed to examine associations between VDR BsmI polymorphism and osteoporosis in postmenopausal women.

## Introduction

Osteoporosis, as a systemic bone disease characterized by decreased bone mineral density, micro-structure deterioration of bone tissue, and increased risk of bone fracture [1, 2], is commonly seen in postmenopausal females and aged males; about 30% of postmenopausal females suffer from osteoporosis [3]. Bone fractures caused by osteoporosis are extremely harmful and are one of the main causes of disability and death in elderly patients. Research on early identification of high-risk groups has been carried out, which is of substantial clinical significance. The pathogenesis of osteoporosis is currently unclear. It is widely accredited that osteoporosis is related to individual genetic differences, estrogen levels, nutritional status, and lifestyle. In addition, osteoporosis can also be induced by bone formation and bone resorption disorder caused by physical injury, diseases affecting bone metabolism, or long-term use of hormone drugs [4].

The interaction between vitamin D and its receptor exerts an important role in calcium homeostasis and bone metabolism by regulating osteocyte growth and differentiation, intestinal calcium absorption, and parathyroid hormone secretion [5]. The vitamin D receptor (VDR) gene is located on chromosome 12 (12q13.1), with a length of more than 100 kb, and more than 100 polymorphic sites are predicted [6, 7]. VDR, therefore, is seen as one of the significant candidate genes to explore the genetic factors leading to osteoporosis. In 1992, Morrison et al. reported that bone mineral density and circulating osteocalcin levels may be affected by VDR BsmI polymorphism (rs1544410) [8, 9].

Postmenopausal osteoporosis, resulting from estrogen deficiency, is the most common type of osteoporosis, and estrogen deficiency results in an increase in bone turnover owing to effects on all types of bone cells [10]. In 1996, Berg et al. reported for the first time that VDR BsmI polymorphism was associated with bone mineral density in postmenopausal females [11]. Since then, epidemiological investigations regarding the assessment of BsmI polymorphism and the susceptibility of postmenopausal osteoporosis have been widely reported. However, the relevant research results have been controversial. For example, in a survey of the Thai population, VDR BsmI polymorphism did not seem to be associated with the risk of postmenopausal osteoporosis [12]. However, significant correlation was observed between VDR genotype and BMD in Chinese postmenopausal females, with bb genotype having the lowest bone density [13]. In recent years, meta-analysis, as a powerful statistical analysis tool, has been adopted to integrate and analyze the data of several published articles; a more accurate and objective assessment is expected to be made on the research results and to explain the heterogeneity between these results [14]. Therefore, the meta-analysis was performed on the currently published eligible case-control studies combined with the previous research results, and the relationship between Bsm I polymorphism and the risk of osteoporosis in postmenopausal females was also explored.

## Methods

### Literature search

PubMed (http://www.ncbi.nlm.nih.gov/pubmed), EMBASE (http://www.embase.com), Weipu (http://www.cqvip.com/), CNKI (http://www.cnki.net/), and Wanfang (http://g.wanfangdata.com.cn/) databases were thoroughly searched by the authors (last search update, July 10, 2020). The keywords were “vitamin D receptor” or “VDR” and “osteoporosis” or “fracture” and “BsmI” or “rs1544410” in combination with “genetic” or “polymorphism” or “variant”.

### Inclusion criteria

Selection criteria of this meta-analysis are listed as follows: ① case-control or cohort studies, ② participants included postmenopausal women, ③ assessment of the relationship of BsmI and osteoporosis or fracture, ④ containing available genotype frequencies of BsmI, ⑤ provided BMD values (mean and standard deviation) of lumbar spine and femoral neck, osteoporosis was defined as BMD ≤ − 2.5 SDs (T-score).

### Exclusion criteria

Exclusion criteria of this meta-analysis are listed as follows: ① reviews, case reports, comments, and letters; ② incomplete data; ③ without full text. In addition, all relevant references were also reviewed. If there were duplicate data in papers published by the same author, only the most recent or complete study was included in this analysis.

### Data extraction

Two independent investigators extracted data from eligible studies; the characteristics included the following: ① the 1st author, ② publication year, ③ region, ④ ethnicity, ⑤ age range, ⑥ sample size, ⑦ allele frequency of cases and controls, and ⑧ genotyping method. Any different evaluation results need to be revisited until a consensus is reached.

### Quality assessment

The quality of eligible publications was assessed by the Newcastle-Ottawa quality assessment scales (NOS) [15]. The scale contains three parts: the selection of groups (4 questions, 1 score each), the comparability of groups (1 question, 2 scores), the ascertainment of exposure (3 questions, 1 score each). The scores ≥ 5 were regarded as a high-quality study.

### Statistics analysis

The observed genotype frequencies of the VDR BsmI polymorphism in control groups were assessed for Hardy-Weinberg equilibrium using the *Χ*^2^ test. The gene frequencies of the control group must conform to the Hardy-Weinberg equilibrium (*p* > 0.05). The relationship between VDR BsmI gene and osteoporosis was accessed by calculating odds ratios (ORs) and 95% confidence intervals (CIs). The pooled ORs were performed for additive genetic model (b vs. B), dominant model (bb + Bb vs. BB), recessive model (bb vs. Bb + BB), and co-dominant model (Bb vs. BB, bb vs. BB) respectively. The subgroup analyses by ethnic groups were also performed. The statistically significant *p* value was set at 0.05. Heterogeneity assumption was evaluated by a chi-square-based *Q* test (*p* < 0.05 indicated heterogeneity across studies). The summary OR estimate of each study was calculated by the fixed-effects model if there was no significant heterogeneity. Otherwise, the random-effects model was used [16, 17]. The potential for publication bias was examined by a Begg’s test (funnel plot method, *p* < 0.05 considered representative of statistical significance) [18]. All analyses were performed by the Stata software (version 11.0).

## Results

### Eligible studies

**Fig. 1:**
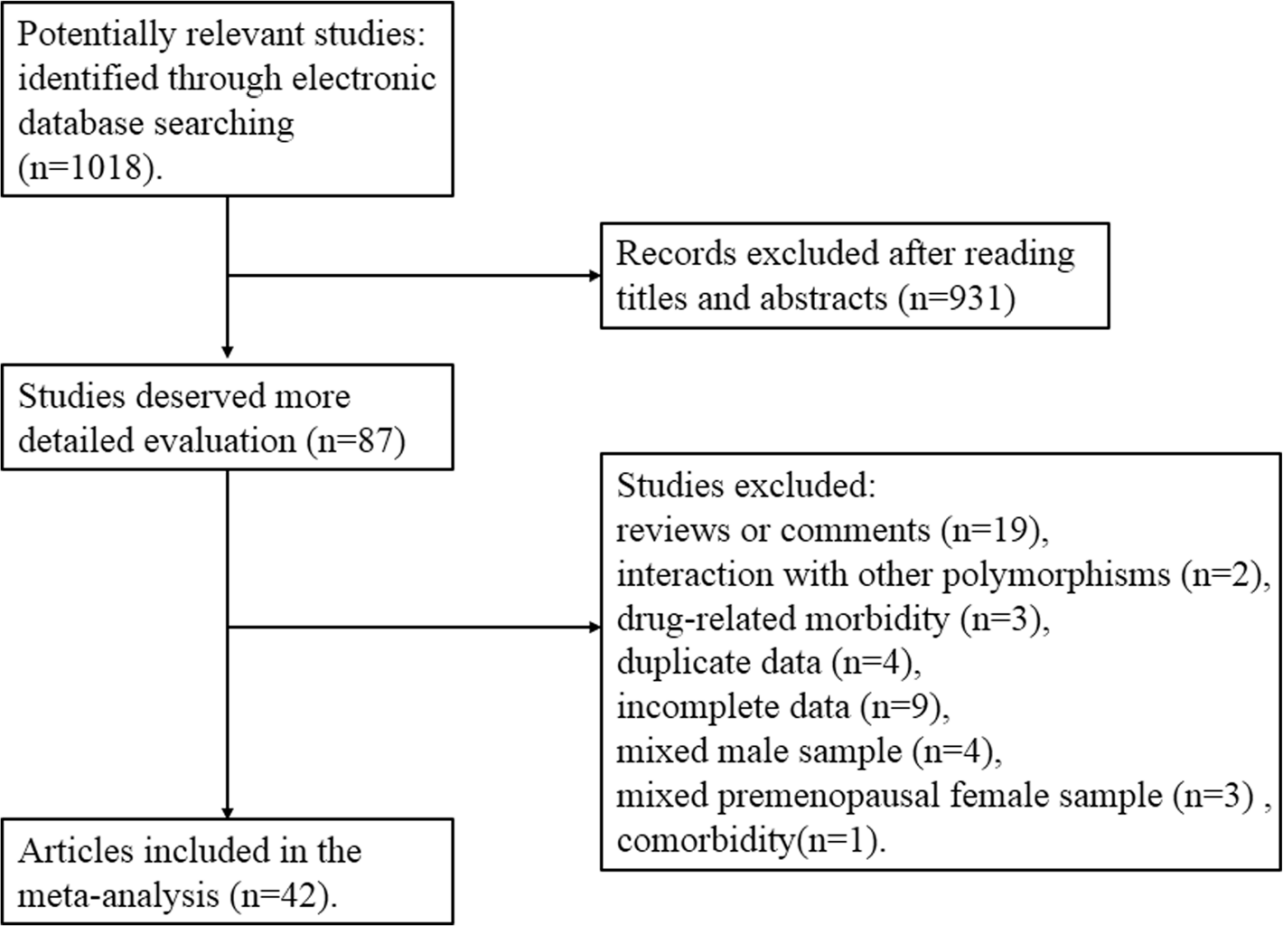
Flow chart indicating search results

Literature screening process is shown in Fig. 1. Based on the pre-established search strategy, 42 studies were finally enrolled for integrated analyses, including 4485 osteoporosis and 5490 controls. Twenty-three studies [19–46] were performed in Caucasians, and 9 studies [12, 13, 47–53] were subsumed into Asians. In addition, 2 interracial studies [32, 54] were conducted in mixed race. The main characteristics of the selected studies are listed in Table 1. According to the NOS for assessing the quality of case-control studies, all the selected articles meet the requirements (the scores ≥ 5, Table 2). The observed genotype frequencies of the VDR BsmI polymorphism in each control group were assessed by Hardy-Weinberg equilibrium (Table 3), and 11 unequal studies were excluded [20, 25, 27, 33, 36–39, 46, 47, 53].

**Table 1:** Main characteristics of studies included in the meta-analysis

BsmI rs1544410 (G > A)	Publication year	Region	Genotyping methods	Osteoporosis		Control	
				***n***	Age (year) mean ± SD	***n***	Age (year) mean ± SD
Marozik et al. [19]	2018	Belarus, Lithuania	PCR-RFLP	149	61.40 ± 6.50	172	57.50 ± 7.30
Ahmad et al. [20]	2018	India	PCR-RFLP	254	55.82 ± 6.91	254	54.76 ± 6.26
Techapatiphandee et al. [12]	2018	Thailand	PCR-RFLP	105	73.10 ± 8.90	132	63.4 0 ± 8.70
Moran et al. [21]	2015	Spain	TaqMan	150	60.24 ± 7.74	30	59.73 ± 9.28
Marozik et al. [22]	2013	Belarus	PCR-RFLP	54	58.30 ± 6.20	77	56.70 ± 7.40
Gonzalez et al. [23]	2013	Mexico	TaqMan	88	57.65 ± 5.58	88	56.34 ± 4.98
Efesoy et al. [24]	2011	Turkey	PCR-RDB	40	65.75 ± 9.80	30	62.40 ± 8.70
Zhang et al. [13]	2011	China	PCR-RFLP	120	60.12 ± 3.26	60	58.69 ± 2.48
Tanriover et al. [25]	2010	Turkey	PCR-RFLP	50	58.30 ± 6.50	50	57.30 ± 6.60
Mansour et al. [26]	2010	Egypt	PCR-RFLP	50	54.40 ± 5.10	20	53.50 ± 5.40
Musumeci et al. [27]	2009	Italy	PCR-RFLP	100	49.91 ± 3.08	100	52.39 ± 4.38
Mencej et al. [28]	2009	Slovenia	PCR-RFLP	240	64.50 ± 8.20	228	61.50 ± 8.30
Seremak et al. [29]	2009	Poland	PCR-RFLP	163	64.27 ± 8.72	63	63.08 ± 7.24
Perez et al. [30]	2008	Argentina	PCR-RFLP	64	62.70 ± 0.86	68	59.40 ± 0.85
Uysal et al. [31]	2008	Turkey	PCR-RFLP	100	---	146	---
Quevedo et al. [32]	2008	Chile	PCR-RFLP	67	77.00 ± 4.00	59	78.00 ± 9.00
Wengreen et al. [33]	2006	USA	PCR-RFLP	819	76.70 ± 9.10	854	76.00 ± 9.40
Garnero et al. [34]	2005	France	PCR-RFLP	120	61.77 ± 8.40	469	61.77 ± 8.40
Mitra et al. [35]	2006	India	PCR-RFLP	119	54.10 ± 3.50	97	54.10 ± 3.50
Duman et al. [36]	2004	Turkey	PCR-RFLP	75	53.16 ± 1.31	66	52.62 ± 1.69
Zhu et al. [47]	2004	China	PCR-RFLP	40	57.55 ± 5.18	158	57.55 ± 5.18
Douroudis et al. [37]	2003	Greece	PCR-RFLP	35	61.37 ± 0.96	44	58.68 ± 1.01
Chen et al. [48]	2003	China	PCR-RFLP	40	54.72 ± 2.60	21	54.72 ± 2.60
Lisker et al. [38]	2003	Mexico	PCR-RFLP	66	65.20 ± 6.80	57	56.50 ± 6.00
Borjas et al. [39]	2003	Venezuela	PCR-RFLP	54	---	55	---
Leng et al. [49]	2002	China	PCR-RFLP	22	51.67 ± 4.93	46	51.67 ± 4.93
A et al. [50]	2002	China	PCR-RFLP	10	53.70 ± 7.11	13	53.70 ± 7.11
Zajickova et al. [40]	2002	Czech	PCR-RFLP	65	63.60 ± 7.80	33	60.10 ± 10.30
Pollak et al. [41]	2001	Israel	PCR-RFLP	75	49.57 ± 2.97	143	49.57 ± 2.97
Valimaki et al. [42]	2001	Finland	PCR-RFLP	372	---	111	---
Aerssens et al. [43]	2000	Belgium	PCR-RFLP	135	78.00 ± 9.00	239	76.00 ± 4.00
Garrofe et al. [44]	2000	Spain	PCR-RFLP	75	58.30 ± 5.00	51	57.20 ± 4.50
Zhang et al. [51]	2000	China	PCR-RFLP	34	66.70 ± 8.50	78	66.70 ± 8.50
Gomez et al. [45]	1999	Spain	PCR-RFLP	37	66.30 ± 8.67	122	63.00 ± 8.67
Ramalho et al. [54]	1998	Brazil	PCR-RFLP	56	78.50 ± 7.20	36	72.90 ± 5.20
Gennari et al. [46]	1998	Italy	Southern Blotting	160	58.20 ± 0.60	144	57.10 ± 0.70
Zhang et al. [52]	1998	China	PCR-RFLP	17	56.76 ± 2.80	162	58.78 ± 3.00
Vandevyver et al. [55]	1997	Belgium	PCR-RFLP		75.50 ± 5.00	698	66.60 ± 8.40
Houston et al. [56]	1996	UK	PCR-RFLP	44	66.00 ± 0.85	44	65.30 ± 0.95
Berg et al. [11]	1996	Norway	PCR-RFLP	19	63-65	30	63-65
Yanagi et al. [53]	1996	Japanese	PCR-RFLP	46	65.00 ± 8.80	66	64.90 ± 6.30
Melhus et al. [1]	1994	Sweden	PCR-RFLP	70	70.00 ± 8.00	76	69.00 ± 8.00

*PCR-RFLP* polymerase chain reaction-restriction fragment length polymorphism. *TaqMan* Taqman probe-based real-time fluorescent quantitative polymerase chain reaction assay in polymorphism, *PCR-RDB* polymerase chain reaction-reverse dot blot, *SD* standard deviation

---: The original text only showed “postmenopausal” and did not provide a specific age range

**Table 2:** Results of quality assessment by the Newcastle-Ottawa Scale for case-control studies

Study	Selection				Comparability	Exposure			Total score
	Adequate definitionof the cases	Representativenessof the cases	Selection ofcontrols	Definition ofcontrols	Control forimportant factors	Ascertainmentof exposure	Same method of ascertainmentfor cases and controls	Non-responserate	
Marozik et al. [19]	√		√	√	√	√	√	√	7
Techapatiphandee et al. [12]	√	√		√	√	√	√	√	7
Ahmad et al. [20]	√	√		√	√√	√	√	√	8
Moran et al. [21]	√				√√	√	√	√	6
Marozik et al. [22]	√			√	√√	√	√	√	7
Gonzalez et al. [23]	√	√		√	√	√	√	√	7
Efesoy et al. [24]	√		√	√	√	√	√		6
Zhang et al. [13]	√			√	√	√	√	√	6
Mansour et al. [26]	√			√	√	√	√	√	6
Tanriover et al. [25]	√	√		√	√√	√	√	√	8
Musumeci et al. [27]	√			√	√	√	√	√	6
Mencej et al. [28]	√	√		√	√	√	√	√	7
Seremak et al. [29]	√			√		√	√	√	5
Perez et al. [30]	√	√	√	√	√	√	√	√	8
Uysal et al. [31]	√			√		√	√	√	5
Quevedo et al. [32]	√		√	√		√	√		5
Wengreen et al. [33]	√	√	√	√	√√	√	√	√	9
Mitra et al. [35]	√	√		√	√	√	√	√	7
Garnero et al. [34]	√			√	√	√	√		5
Duman et al. [36]	√			√	√√	√	√	√	7
Zhu et al. [47]	√		√	√	√	√	√	√	7
Douroudis et al. [37]	√			√	√	√	√	√	6
Borjas-Fajardo et al. [39]	√			√		√	√	√	5
Chen et al. [48]	√		√	√		√	√	√	6
Lisker et al. [38]	√	√		√	√	√	√	√	7
Zajickova et al. [40]	√	√		√	√	√	√	√	7
Leng et al. [49]	√		√	√		√	√	√	6
Ly et al. [50]	√		√			√	√	√	5
Pollak et al. [41]	√			√		√	√	√	5
Valimaki et al. [42]	√		√	√		√	√		5
Aerssens et al. [43]	√	√	√	√	√	√	√	√	8
Garrofe et al. [44]	√		√	√	√	√	√	√	7
Zhang et al. [51]	√			√	√	√	√	√	6
Gennari et al. (1999)	√	√		√	√	√	√	√	7
Gomez et al. [45]	√			√	√√	√	√		6
Ramalho et al. [54]	√			√	√√	√	√	√	7
Gennari et al. [46]	√		√	√	√√	√	√	√	8
Zhang et al. [52]	√		√	√		√	√	√	6
Vandevyver et al. [55]	√			√	√√	√	√	√	7
Houston et al. [56]	√				√√	√	√	√	6
Berg et al. [11]	√			√	√	√	√		5
Yanagi et al. [53]	√		√	√		√	√		5
Melhus et al. [1]	√		√			√	√	√	5

√: matched the condition, scored one point

**Table 3:** The distribution of VDR BsmI genotypes for postmenopausal osteoporosis and controls

BsmI rs1544410 (G > A)	Publication year	Ethnicity	Osteoporosis			Control			***p***^**a**^
			BB	Bb	bb	BB	Bb	bb	
Marozik et al. [19]	2018	Caucasian	53	64	32	35	73	64	0.098
Ahmad et al. [20]	2018	Caucasian	54	137	63	54	152	48	**0.002**
Moran et al. [21]	2015	Caucasian	18	65	67	3	19	8	0.097
Marozik et al. [22]	2013	Caucasian	12	31	11	11	26	40	0.061
Gonzalez et al. [23]	2013	Caucasian	54	28	6	46	38	4	0.267
Efesoy et al. [24]	2011	Caucasian	5	23	12	5	15	10	0.876
Tanriover et al. [25]	2010	Caucasian	15	19	16	19	7	24	**< 0.001**
Mansour et al. [26]	2010	Caucasian	27	15	8	1	2	17	0.050
Musumeci et al. [27]	2009	Caucasian	30	55	15	13	60	27	**0.025**
Mencej et al. [28]	2009	Caucasian	103	110	27	88	100	40	0.215
Seremak et al. [29]	2009	Caucasian	27	66	70	10	27	26	0.506
Perez et al. [30]	2008	Caucasian	17	35	12	20	32	16	0.647
Uysal et al. [31]	2008	Caucasian	18	48	34	24	78	44	0.283
Quevedo et al. [32]	2008	Caucasian	11	46	10	9	37	13	0.050
Wengreen et al. [33]	2006	Caucasian	154	393	272	140	376	338	**0.043**
Garnero et al. [34]	2005	Caucasian	25	62	33	65	224	180	0.724
Mitra et al. [35]	2006	Caucasian	51	46	22	19	38	40	0.080
Duman et al. [36]	2004	Caucasian	18	54	3	17	42	7	**0.014**
Douroudis et al. [37]	2003	Caucasian	3	12	20	10	29	5	**0.026**
Lisker et al. [38]	2003	Caucasian	15	17	34	13	38	6	**0.008**
Borjas et al. [39]	2003	Caucasian	28	20	6	11	36	8	**0.020**
Zajickova et al. [40]	2002	Caucasian	21	24	20	10	13	10	0.223
Pollak et al. [41]	2001	Caucasian	13	38	24	16	67	60	0.675
Valimaki et al. [42]	2001	Caucasian	44	175	153	20	55	36	0.899
Aerssens et al. [43]	2000	Caucasian	26	60	49	52	125	62	0.459
Garrofe et al. [44]	2000	Caucasian	9	49	17	10	22	19	0.434
Gomez et al. [45]	1999	Caucasian	7	20	10	20	51	51	0.241
Gennari et al. [46]	1998	Caucasian	40	92	28	11	76	57	**0.035**
Vandevyver et al. [55]	1997	Caucasian	12	50	24	127	368	203	0.076
Houston et al. [56]	1996	Caucasian	8	19	17	9	19	16	0.450
Berg et al. [11]	1996	Caucasian	4	8	7	8	11	11	0.156
Melhus et al. [1]	1994	Caucasian	14	29	27	7	35	34	0.637
Techapatiphandee et al. [12]	2018	Asian	85	19	1	103	25	4	0.123
Zhang et al. [13]	2011	Asian	9	25	86	16	36	8	0.086
Zhu et al. [47]	2004	Asian	6	26	8	7	105	46	**< 0.001**
Chen et al. [48]	2003	Asian	0	7	33	0	3	18	0.724
Leng et al. [49]	2002	Asian	0	11	11	7	19	20	0.488
Ly et al. [50]	2002	Asian	0	4	6	1	5	7	0.935
Zhang et al. [51]	2000	Asian	2	15	17	7	36	35	0.598
Zhang et al. [52]	1998	Asian	0	3	14	0	14	148	0.565
Yanagi et al. [53]	1996	Asian	12	12	22	2	7	57	**0.013**
Ramalho et al. [54]	1998	mix	13	23	20	7	11	18	0.050
Quevedo et al. [32]	2008	mix	11	46	10	9	37	13	0.050

The bold values emphasize that the data does not conform to the Hardy–Weinberg equilibrium, to facilitate the readers to scan the content

^a^ *p* value for Hardy–Weinberg equilibrium in the control group

### Meta-analysis

**Fig. 2:**
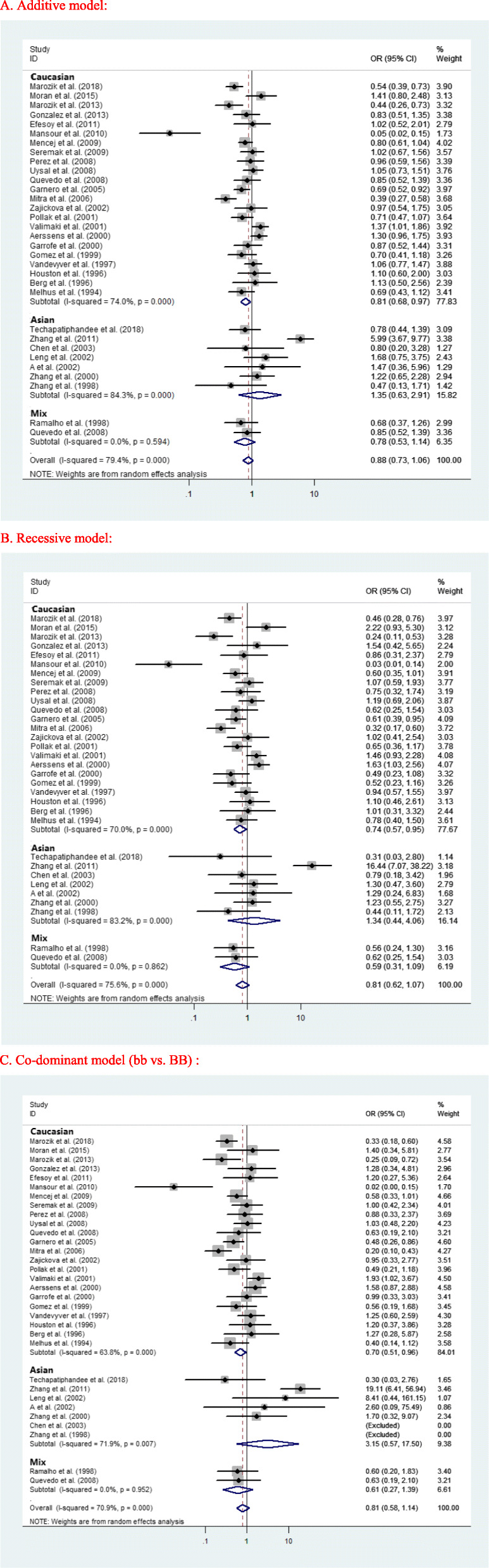
Association of VDR BsmI polymorphism under different genetic models with osteoporosis risk in postmenopausal women. **a** Additive model. **b** Recessive model. **c** Co-dominant model (bb vs. BB)

Differences in allelic distribution by ethnicity could be partially responsible for the observed differences in the association between VDR BsmI and osteoporosis. The evaluations of the association between VDR Bsml polymorphism and osteoporosis risk in postmenopausal women are summarized in Table 4. The overall results suggested that there was no association between BsmI polymorphism and the risk of osteoporosis in all genetic models. In the subgroup analysis based on ethnicity, the included studies were divided into Asian, Caucasian, and mix populations. The results showed that VDR BsmI polymorphism was significantly associated with the risk of postmenopausal osteoporosis in Caucasian populations (additive model: OR 0.809, 95% CI 0.678~0.965, *p* = 0.019; recessive model: OR 0.736, 95% CI 0.568~0.955, *p* = 0.021; and co-dominant model: bb vs. BB OR 0.701, 95% CI 0.511~0.962, *p* = 0.028, Fig. 2). However, no significant association was found in any genetic models in both Asian and mix populations.

**Table 4:** ORs and 95% CI for postmenopausal osteoporosis and the VDR BsmI polymorphism under different genetic models

Genetic model	Population	Pooled OR [95% CI] ***p***	Heterogeneity ***p*** value*	Publication bias
				Begg’s test ***p*** value
Additive (b vs. B)	Caucasian	0.809 [0.678~0.965] **0.019**	< 0.001	0.893
	Asian	1.353 [0.628~2.915] 0.440	< 0.001	0.881
	Mix	0.778 [0.530~1.144] 0.202	0.594	0.317
	overall	0.880 [0.729~1.063] 0.185	< 0.001	0.856
Recessive (bb vs. Bb/BB)	Caucasian	0.736 [0.568~0.955] **0.021**	< 0.001	0.853
	Asian	1.340 [0.442~4.061] 0.605	< 0.001	0.652
	Mix	0.585 [0.314~1.090] 0.091	0.862	0.317
	overall	0.813 [0.619~1.066] 0.134	< 0.001	0.815
Dominant (Bb/bb vs. BB)	Caucasian	0.810 [0.654~1.004] 0.055	0.009	0.833
	Asian	2.107 [0.768~5.784] 0.148	0.033	0.806
	Mix	0.860 [0.426~1.736] 0.673	0.848	1
	overall	0.884 [0.715~1.092] 0.253	0.001	0.813
Bb vs. BB	Caucasian	0.880 [0.754~1.027] 0.105	0.427	0.579
	Asian	1.206 [0.738~1.969] 0.455	0.627	0.117
	Mix	1.061 [0.501~2.248] 0.878	0.896	0.317
	overall	0.911 [0.788~1.053] 0.206	0.615	0.510
bb vs. BB	Caucasian	0.701 [0.511~0.962] **0.028**	< 0.001	0.895
	Asian	3.146 [0.566~17.50] 0.190	0.007	0.117
	Mix	0.612 [0.270~1.391] 0.241	0.952	0.317
	overall	0.811 [0.576~1.141] 0.228	< 0.001	0.937

*CI* confidence interval

* *p* value for heterogeneity test; random-effects model was used when *p* value for heterogeneity test < 0.05

### Publication bias

Begg’s test was performed to quantitatively evaluate the publication bias of literatures on osteoporosis. The results provided statistical evidence in overall results, suggesting the absence of publication bias. All graphical funnel plots of the included studies appeared to be symmetrical. There was no visual evidence of publication bias visually from the funnel plot, which implied that the publication bias was low in the present overall meta-analysis (b vs. BB: *p* = 0.856; b/b vs. Bb/BB: *p* = 0.851; Bb/bb vs. BB: *p* = 0.813; Bb vs. BB: *p* = 0.510; bb vs. BB *p* = 0.937).

## Discussion

Genetic difference is one important factor affecting the susceptibility to osteoporosis. VDR gene has been widely studied because of its important role in regulating bone metabolism and bone homeostasis. The VDR Bsml polymorphism is located in the 3′ untranslated region (UTR). It is involved in regulating the stability of VDR mRNA and is one of the most important subtypes of VDR gene polymorphism. Studies on VDR Bsml polymorphism and susceptibility to osteoporosis are various, but the results are not consistent. A recent meta-analysis [57] shows that VDR BsmI is associated with an increased risk of postmenopausal osteoporosis in Asians, while in Caucasians seem to be unrelated, which is contrary to the results of two previously published studies [58, 59]. Since the previous meta-analysis only involved genetic association studies published before 2015, the combination of different original data in each study might have a great impact on the mixed distribution of genotypes. So introducing new data to update meta-analysis is necessary. Through our meta-analysis, it has been found that the VDR Bsml gene polymorphism generally seems not to be a susceptibility gene for postmenopausal osteoporosis. However, in the subgroup analysis, BsmI polymorphism was found to be associated with the risk of postmenopausal osteoporosis in Caucasians, which was not found in the previous meta-analysis. In Asian postmenopausal women, there was no obvious relationship between Bsml polymorphism and osteoporosis susceptibility, which was consistent with the results of a previous meta-analysis of the Chinese population [60]. Through sensitivity analysis and publication bias detection, the results of this meta-analysis were true and credible. The original data of all published eligible studies were almost covered by this meta-analysis. However, according to a recently published meta-analysis of Yadav et al. [61], in the absence of a subgroup analysis based on the sex and age of patients or the type of osteoporosis, BsmI polymorphism seemed not to be associated with the pathogenesis of osteoporosis. It indicated that possible relationship between VDR gene polymorphisms and osteoporosis may be related to gender, race, and age difference of subjects. There may be different mechanisms of VDR gene polymorphisms on different types of osteoporosis [62]. Our research also verified this point, and it should be regarded as a valuable supplement to the published related studies. The causes of osteoporosis are complex; in addition to the joint effects of multi-gene regulation, environmental factors and lifestyles also play an important role [63]. This meta-analysis only discussed genetic factors in the original literature, and the interaction of other factors such as dietary calcium and light exposure and VDR gene polymorphisms on osteoporosis was uninvolved. Therefore, referring to the analysis methods of other researchers [64], we focused on checking the details of the dietary habits of the participants in each study and determined that there was no difference in calcium intake between the case-control group. We believe that for more accurate evaluation of the relationship between vitamin D receptor gene polymorphism and postmenopausal osteoporosis, researches having large samples are required, and the synergy of other factors such as diet, environment, and exercise should be considered more comprehensively when cases are included in the group.

## Conclusions

In conclusion, our study believes that VDR BsmI polymorphism and postmenopausal osteoporosis are genetically linked in Caucasians, but not in Asians. It is necessary to conduct large-scale studies to verify the correlation of different populations and environmental factors in the susceptibility to osteoporosis.

## Data Availability

Not applicable.
